# Role of Genetic Changes in the Progression of Cardiovascular Diseases

**Published:** 2011-12

**Authors:** S. A. Sheweita, H. Baghdadi, A. R. Allam

**Affiliations:** 1 *Department of Clinical Biochemistry, College of Medicine, Taibah University, Saudi Arabia;*; 2 *Departments of Medicine, College of Medicine, Taibah University, Saudi Arabia*

**Keywords:** cardiovascular diseases, genetic polymorphisms of nitric oxidesynthase, hyperlipidemia, hyperhomocysteinemia polymorphisms, paraoxonase polymorphisms

## Abstract

This review aims to investigate the role of genetic changes in the development of cardiovascular diseases [CVD]. Oxidation of Low density Lipoprotein (LDL) and mutations in LDL receptors gene are a trigger for numerous of atherogenic events. Also, endothelial nitric oxide synthase *(eNOS)* plays an important role in vasodilatation of blood vessels through synthesis of nitric oxide. Three single base pair changes, 786T/C, 922A/G, and 1468T/A, have been identified in the promoter region of the *eNOS* gene and are associated with coronary spasm. Moreover, two distinct variable nucleotide tandem repeats (VNTRs) in introns 4 and 13 have been detected. The presence of a minimum of 38 CA repeats in intron 13 has been associated with an independent 2.2-fold increase in the risk of coronary artery disease [CAD]. Plasma glutathione peroxidase (*GPx-3*) maintains the vascular bioavailability of nitric oxide (NO), through depletion of reactive oxygen species. Mutation(s) or polymorphism(s) in the plasma *GPx-3* gene promoter may predispose to a thrombotic disorder, and constitute a genetic risk factor for thrombotic cerebrovascular disease. Hyperhomocysteinemia is another independent risk factor for atherosclerosis and arterial thrombosis. Severe hyperhomocysteinemia could be caused by cystathionine-β-synthase enzyme deficiency but it could be due to homozygosity of a common 677C/T point mutation in the coding region of the methylenetetrahydrofolate reductase (MTHFR) gene as a 3-fold increase in risk of CAD is associated with the MTHFR *677TT* genotype. A second common variant in MTHFR 1298A/C is associated with decreased enzyme activity *in vitro* and *in vivo*, especially when occurring simultaneously with the 677 C/T polymorphism. Elevated fibrinogen, an essential component of the coagulation system, has been most consistently associated with arterial thrombotic disorders. Several polymorphisms (148C/T, 455G/A, and -854G/A) have been identified in the genes encoding the 3 pairs of fibrinogen polypeptide chains. The -455G/A, and -854G/A substitutions are the most physiologically relevant mutations. In addition the -*455A* allele has been associated with the progression of atheroma, and also with a 2.5-fold increase in risk of multiple lacunar infarcts in a cohort of elderly patients with stroke. It is concluded that genetic changes in the previously mentioned genes could play a significant role in the initiation and progression of CVD. This review provides useful information for both physicians and medical students whom are interested in human genetics which is related to cardiovascular diseases.

## INTRODUCTION

More than 1.5 million individuals have a myocardial infarction each year and coronary artery disease (CAD) is responsible for about 500,000 deaths with coronary atherosclerosis being the major cause of CAD (1, 2). The atherosclerotic process is an organized, active, lifelong process involving elements of chronic inflammation followed by repair in the artery wall (3, 4). The endothelium and many growth factors, cytokines, and vasoregulatory molecules are involved in the atherosclerotic process (3). Atherosclerosis begins with the appearance of fatty streaks in the intimae (5). The fatty streaks can then be transformed into fibrous plaques by accumulation of lipid in smooth muscle and connective tissue. These plaques undergo vascularization, intraplaque hemorrhage, rupture, ulceration, and calcification thereby impeding arterial blood flow. Superimposition of thrombosis on a lesion can lead to myocardial infarction or sudden death (5, 6). Comprehensive studies to date have identified risk factors for cardiovascular diseases (CVD) which include older age, male sex, elevated total cholesterol levels, elevated low-density lipoprotein cholesterol levels, reduced high-density lipoprotein cholesterol levels, hypertension, diabetes mellitus, smoking, obesity, physical inactivity, and genetic changes in some enzymes (7-9). In this review, we are focusing on the genetic changes of genes which contribute to cardiovascular diseases susceptibility.

### Genetics changes as risk factors affecting cardiovascular diseases

**Genetics of Dyslipidemia.** Serum cholesterol concentrations are profoundly influenced by the composition of dietary fat, with saturated fatty acids (SFAs) being the major determinant of serum cholesterol (10, 11) There are wide interindividual variations in total, LDL, and HDL cholesterol and lipoprotein responses (12-14). The existence of consistent hypo- and hyperresponders supports the hypothesis that the degree of responsiveness is related to genetic variation (15-17). Single-gene disorders are the most dramatic effects of the genetic contributions to atherosclerosis (9, 18, 19). However, most forms of the CVD are the product of many gene changes with small environmental effects. Cardiovascular disease has a multifactorial etiology with many established risk factors (20). Many studies have investigated this possibility and have largely focused on genes whose products affect lipoprotein metabolism, e.g. apolipoproteins, enzymes, and receptors (20).

In the last few years, much has been added to our understanding of the genetic basis of dyslipidemias and the role of different molecular players in the pathogenesis of these disorders. New information has also emerged regarding the efficacy and safety of intensive lipid-lowering therapies from many large-scale randomized clinical trials (21). Hypercholesterolemia as well as hypertriglyceridemia in the vast majority of patients are either secondary to diet, obesity, medications, or other disorders affecting lipoprotein metabolism, or are polygenic in origin. All the known genes defective in patients with monogenic hypercholesterolemias are involved in the receptor-mediated uptake of low-density lipoproteins (LDL) by the LDL receptor (LDLR) in hepatocytes (22, 23). Autosomal dominant familial hypercholesterolemia (FH) has been well recognized to be due to mutations in *LDLR* gene and is one of the most common inherited metabolic diseases (22). Previous studies revealed that *LDLR* mutations have been reported in 52-76% of patients meeting the clinical criteria for monogenic hypercholesterolemia (24). Loss of function or reduced LDLR number in the hepatocytes results in reduced clearance of plasma LDL and a 2- to 3-fold elevation in LDL cholesterol levels in heterozygous FH patients. Approximately half of these patients develop tendon xanthomas, xanthelasmas, premature corneal arcus, and coronary heart disease (CHD) which occurs in the fourth or fifth decades of life. Homozygous or heterozygous patients have a greater than 5-fold increase in plasma LDL cholesterol levels and often develop severe atherosclerosis before the age of 20 years (25).

Familial defective apolipoprotein B, an autosomal codominant disorder due to mutations in apolipoprotein B (*APOB*) gene, is indistinguishable from FH due to *LDLR* mutations. Certainly, the frequency of *APOB* mutations in patients with hypercholesterolemia phenotype is reported to be much less than *LDLR* mutations and varies from 0-11% in 40-year-old subjects in Slovakia (26). Currently, eight more missense mutations in the LDLR binding domain of the APOB have been identified (27-29). Previous studies revealed that familial defective apolipoprotein B-100 [FDB] patients have 20-25% lower LDL cholesterol levels compared with FH patients with *LDLR* mutations (30), and have lower risk of CHD (31). Recently, variants in LDLR gene have been classified as pathogenic and non-pathogenic in familial hypercholesterolemia [FH] (32). They have found that 862G>A, and 895G>A variants are causative of FH and pathogenic in the Spanish population (Etxebarria *et al*., 2011). In another study, LDLR gene mutations: 81T>G, 1775G>A, 517T>C, 858C>A, 1352T>C, 1285G>A, 761A>C, 1195G>A, 1646G>A, and a deletion mutation in exon 4 have been detected in 164/254 Greek patients (33). In addition, no functional mutations in ARH, Apo B and PCSK9 genes have been detected in 40 patients with no LDLR gene defect (33). Moreover, Twenty-five mutations (24 LDLR, 1 APOB) were identified in 38/62 Mexico patients (34) and also a novel PCSK9 mutation, c.1850 C>A (p.Ala617Asp) was detected (34). In France, mutations in the LDLR gene were identified in 1003 ADH subjects representing 391 unique events with 46.0% missense, 14.6% frameshift, 13.6% splice, and 11.3% nonsense mutations, 9.7% major rearrangements, and 3.8% small in frame deletions/insertions mutations (35). Furthermore, mutations in the APOB gene were identified in 89 probands and in the PCSK9 gene in 10 probands. The respective contribution of each known gene to ADH in French cohort was: LDLR 73.9%, APOB 6.6%, PCSK9 0.7% (35).

Proprotein convertase subtilisin-like kexin type 9 (*PCSK9*) has been involved in autosomal dominant hypercholesterolemia since PCSK9 binds and favors degradation of the low-density lipoprotein receptor (LDLR) and thereby modulates the plasma levels of LDL-cholesterol. Interestingly, some loss-of-function in *PCSK9* variants has been reported to be associated with low levels of plasma LDL cholesterol and reduced risk of CHD (36, 37, 38). The clinical features of PCSK9 mutations in these patients are also similar to FH patients with *LDLR* mutations (39). LDLR adaptor protein (LDLRAP1 or ARH) promotes the clustering of LDLRs into the clathrin-coated pits on the basolateral surface of hepatocytes by coupling the cytoplasmic tail of LDLR to structural components of the clathrin coated pit (40) and thus is essential for LDLR-mediated endocytosis. Inactivating mutations in LDLRAP1 lead to retention of LDLRs on the apical surface, thereby severely reducing LDL uptake. Serum LDL cholesterol levels in ARH patients have been reported to be lower or similar to those seen in patients with homozygous *LDLR* mutations (41), but no differences were seen in the prevalence of planar, tuberous, or tendon xanthomas. Interestingly, ARH patients had markedly reduced prevalence of CHD compared with those with homozygous *LDLR* mutations (41). Another rare autosomal recessive disorder in which elevations of serum cholesterol might be observed along with markedly elevated plasma levels of plant sterols is sitosterolemia or alternatively called phytosterolemia. Mutations in two adjacent ATP-binding cassette transporters, ABCG8 (sterolin-2) and ABCG5 (sterolin-1) that regulate sterol transport at the apical surface of hepatocytes and enterocytes have been identified in patients with sitosterolemia (42, 43). ABCG5 and ABCG8 dimerize to form a functional complex necessary for efflux of dietary cholesterol and noncholesterol sterols from the intestine and liver (44). Thus, mutations in either of these two genes lead to increased absorption and accumulation of cholesterol and plant sterols.

**Genetic polymorphisms of paraoxonase.** Human paraoxonase/arylesterase (PON-1) (EC3.1.1.2) is a calcium-dependent glycoprotein that is present bound to HDL particles (45). Paraoxonase-1 (PON1) is an HDL-associated protein of 354 amino acids with a molecular mass of 43 000 Da. It is synthesized in the liver, and in serum is almost exclusively associated with HDL (46). The paraoxonase (PON) gene family is composed of three members (PON1, PON2, PON3) that share considerable structural homology and are located adjacently on chromosome 7 in humans (47). By far the most-studied member is PON1, a high-density lipoprotein-associated esterase/lactonase, also endowed with the capacity to hydrolyze organophosphorus insecticides, but all the three proteins prevent oxidative stress and fight inflammation (48). High-density lipoprotein (HDL) is an independent protective factor against cardiovascular disease (45). The enzyme paraoxonase-1 (PON-1) contributes to the antiatherogenic effects of HDL. The antiatherogenic potential of paraoxonase is derived from its capacity to hydrolyze oxidized lipids, phospholipids, and cholesterol ester hydroperoxides, thus preventing them from accumulating in LDL particles (45).

Oxidized LDL has been reported to trigger a number of putatively atherogenic events including the induction of proinflammatory molecules that lead to an increase in recruitment of inflammatory cells to the artery wall (46). HDL, on the other hand, is antiatherogenic, and its protective effects have been ascribed primarily to its ability to shuttle excess cholesterol from peripheral tissues to the liver in the reverse cholesterol transport pathway and also to its capacity to protect against LDL oxidation (47, 48). These protective effects of HDL have been attributed to the presence of various proteins associated with HDL in the circulation including apolipoprotein AI, lecithin cholesteryl-acyl transferase (LCAT), and serum paraoxonases (PONs). The activity of plasma PON was lower in individuals with PON1 55 LM genotype than those with LL genotype (49). Gene polymorphisms of PON1 55 Met/Leu, PON2 148 Ala/Gly are involved in the morbidity of CHD by influencing the plasma activities of PON (49, 50). As a result, research in these fields has blended, with the goal of further elucidating the role of this polymorphic enzyme in disease and in an individual’s response to exogenous agents. Four additional polymorphisms have been found in the 5’-regulatory region of PON1 (51), but they have a lesser effect on PON1 protein levels (52). Complete re-sequencing of PON1 from several individuals has led to the identification of nearly 200 new single nucleotide polymorphisms, some in the coding regions and others in introns and regulatory regions of the gene (51). Though most of the new polymorphisms have not yet been characterized, a few have already provided explainations for discrepancies found when comparing PON1 status and PCR analysis of codon 192 (51). Variation in serum PON1 activity by its polymorphisms in both the coding and the 5’-regulatory regions of PON1 gene should also be considered (53, 54). A functional genomic analysis, however, provides a much more informative approach, as direct measurement of an individual’s PON1 function (plasma activity) takes into account all polymorphisms that might affect activity.

**Genetic polymorphism of endothelial nitric oxide (NO) synthase.** Nitric oxide (NO), a product of the normal endothelium, has a variety of physiologic effects that converge to maintain normal endothelial function and an antithrombotic intravascular milieu. NO is a smooth muscle relaxant and inhibits the adhesion, activation, and aggregation of platelets (55). NO-dependent endothelial dysfunction is now accepted as a key initial step in atherothrombogenesis. NO is produced by the constitutive endothelial isoform of the nitric oxide synthases (NOSs) as a by-product of the conversion of L-arginine to L-citrulline (56). The endothelial NOS gene (*eNOS*) is located on chromosome 7q35-36 and comprises 26 exons (57). Numerous polymorphic sites have been identified in the *eNOS* gene, most of which are intronic. Two distinct variable nucleotide tandem repeats (VNTRs) in introns 4 and 13 have been examined in association with vascular disease. The intron 4 VNTR is characterized by the presence of either 4 (minor allele) or 5 (major allele) copies of a 27-bp repeat. A mild but significant reduction in plasma levels of nitrogen oxides had been observed in homozygotes of the minor allele (58). In intron 13, between 17 to 44 copies of a CA repeat have been described, and the presence of a minimum of 38 repeats has been associated with an independent 2.2-fold increase in the risk of CAD (59). Three strongly linked, single base pair changes, 786T/C, 922A/G, and 1468T/A, have been identified in the promoter region of the *eNOS* gene and associated with coronary spasm in the Japanese population (60). The authors found that the substitution at position 786T/C resulted in a significant reduction in *eNOS* gene promoter activity, whereas the other 2 polymorphisms had no effect. Japanese carriers of the *786CC* genotype have also been shown to have reduced cerebral blood flow, yet this effect was only observed among smokers (61). Two Italian studies found an association of the *786C* allele with angiographically defined CAD, as well as with endothelial dysfunction among hypertensive individuals (62). However, numerous other studies have failed to confirm these observations. A G/T base-pair change at position 894 in exon 7 predicts a Glu298Asp substitution, which is the only polymorphism that alters the primary structure of the protein (63). This amino acid change influences enzyme stability, the 298Asp isoform being degraded more rapidly than its 298Glu counterpart. Numerous studies have identified the role for this polymorphism in atherothrombotic disease and risk of myocardial infarction (64-66).

**Polymorphisms of Glutathione Peroxidase (*GPx-3*) Gene.** There are 5 known isoforms of glutathione peroxidase [GPx]. Glutathione peroxidase (GPx-3) is a major antioxidant enzyme in plasma and scavenges reactive oxygen species (ROS) such as hydrogen peroxide and organic (lipid) hydroperoxides produced during normal metabolism or after oxidative insult (67, 68). Nitric oxide (NO), a major vasorelaxant and inhibitor of platelet function, can be inactivated rapidly by ROS. Therefore, GPx-3 contributes significantly to maintain the vascular bioavailability of nitric oxide level. In addition, the reduction of oxidant stress by GPx-3 activity also protects against post-translational modifications of fibrinogen by ROS and NO-derived oxidants that increase its thrombogenicity (69, 70). Two studies of families with idiopathic childhood stroke have provided clinical evidence for the importance of GPx-*3* in the modulation of NO bioavailability and thrombosis (71, 72). The patients in these studies had hyperreactive platelets, and their plasma impaired the normal inhibition of platelet activation by NO. These findings were determined to be a consequence of a familial reduction in GPx-*3* activity that correlated with decreased protein expression of this enzyme (73). Consistent with this role, functional transcription site of the GPx-3 gene showed that it is regulated by oxygen tension and redox state (73).Therefore, it is hypothesized that mutation(s) or polymorphism(s) in the plasma GPx-*3* gene promoter may be responsible for the reduction in enzyme activity and predispose to a thrombotic disorder, thus constituting a genetic risk factor for thrombotic cerebrovascular disease. Supporting this hypothesis, it has been found that direct sequencing of GPx-*3* promoter fragments obtained from CVT patients and controls revealed the presence of 8 promoter polymorphisms (-942 A3C, -927 T3C, -861A3T, -302 A3T, -284 T3A, -568 T3C, -518 T3C, and -65 T3C (74-77). A small series of reports has demonstrated decreased GPx-*3* activity among patients with CAD (78, 79). Taken together, this evidence points to the GPx-*3* gene as a candidate gene for cardiovascular disease risk.

Another isoform of glutathione peroxidases is called GPx1, the key antioxidant enzyme in vascular endothelial cells and plays a protective effect against the presence of coronary artery disease [CAD] (80). It has been found that subjects with 198Pro/leu and 198Leu/leu variants genotype had a significantly higher risk of CAD compared to the 198Pro/Pro carriers in Chinese population (80). In addition, it has been found that the presence of Pro197Leu substitution of the GPx-1 gene may play a crucial role in determining genetic susceptibility to coronary-arteriosclerosis in type II diabetic patients (81).

**DNA alterations in glutathione S-transferase (GSTM1 genotype).** The glutathione (GSH) system is a major player in protection against a broad variety of toxic agents involved in the pathogenesis of several chronic degenerative diseases (82, 83). Most of the biological functions of this tripeptide (γ -glutamyl-L-cysteinyl glycine) depend on the reactivity of the thiol group of its cysteinyl residue (84). The reaction rate of GSH with electrophiles is mainly dependent on GSH-S-transferases (GST), which catalyze conjugation processes resulting in detoxification and excretion of water-soluble conjugates (85, 86). Genetic polymorphisms have been detected within this broad family of phase II drug metabolism isoenzymes. In particular, two of these encoding genes, identified as *GSTM1* (m) and *GSTT1* (u), have a null genotype in humans due to the deletions of both paternal and maternal alleles, resulting in lack of active proteins (87). Polymorphisms within *GSTT1* and especially *GSTM1* have been associated with cancer in various organs (88, 89). In addition, the null *GSTM1* genotype has been associated with elevated levels of DNA adducts, which are promutagenic and procarcinogenic lesions, in leukocytes, bronchi, and lung tissue (90, 91). DNA adducts have consistently been demonstrated in smooth muscle cells (SMC) of human abdominal aorta affected by atherosclerotic lesions (92). All of the surgical samples analyzed contained DNA adducts as measured by ^32^Ppost labeling; and these adduct levels were correlated significantly with known atherogenetic risk factors including age, number of currently smoked cigarettes, arterial pressure, blood cholesterol (total/high density lipoproteins), triglycerides, and oxidative DNA damage in the same cells (92). These findings were further supported by a study evaluating 30 thoracic aortic samples taken at autopsy; DNA adduct levels were significantly correlated with total and low density lipoproteins and cholesterol and were higher in subjects with frequent atherosclerotic changes as compared to subjects with rare lesions (93).

**Glucose-6-phosphate Dehydrogenase.** Nitric oxide (NO) has been shown to modulate angiogenesis by mediating growth factor-stimulated endothelial cell migration and proliferation (94). NO is permissive for endothelial cell migration and enhances directional migration by inducing a switch from a stationary to a mobile phenotype (95, 96). In addition, NO promotes endothelial cell proliferation, and further, proliferating endothelial cells demonstrate increased expression of the endothelial isoform of nitric-oxide synthase (eNOS) compared with quiescent cells (97). Glucose-6-phosphate dehydrogenase (G6PD), the first and rate-limiting enzyme in the pentose phosphate pathway, is the principal intracellular source of NADPH which in turn, is utilized directly as a cofactor for eNOS and, indirectly serves to maintain levels of another important cofactor, tetrahydrobiopterin, via de novo synthesis and the dihydrofolate reductase salvage pathway. In this manner, G6PD regulates both eNOS activity and NO levels (98). The association between G6PD activity and cell proliferation has been well described in several non-vascular cell types (99, 100). Growth factors, such as platelet-derived growth factor and epidermal growth factor, have been shown to increase cell proliferation by increasing both basal G6PD activity and G6PD expression (99). Adenoviral gene transfer of G6PD increases G6PD expression 2-3-fold in COS-7 cells and results in a marked increase in (^3^H)thymidine incorporation (99). Similarly, NIH 3T3 cells transfected with human G6PD cDNA exhibited contact- and anchorage-independent growth (101). These findings demonstrated that G6PD activity was associated with cell proliferation in non-vascular cell lines. New evidence indicates that G6PD activity is associated with vascular endothelial cell proliferation. The mechanism by which G6PD enhances acquisition of an angiogenic phenotype has been characterized (102). G6PD activity modulates endothelial cell migration, proliferation, and tube formation by mediating NO levels. Therefore, G6PD serves as a novel regulatory determinant of the angiogenic phenotype (102). It has been demonstrated that intracellular NADPH levels, and not the production of ribose moieties by G6PD, are the critical determinant of cell growth (99). This suggests that one mechanism by which G6PD regulates endothelial cell migration, proliferation and tube formation is by synthesizing NO via eNOS, which has an absolute requirement for NADPH as a cofactor. The relationship between G6PD and NOS activity has been demonstrated in several cell lines. For example, NO production is impaired in G6PD-deficient granulocytes stimulated with lipopolysaccharide or phorbol 12-myristate 13-acetate. Interestingly, this response resulted from a decrease in activity, not expression, of the inducible form of NOS (103). In vascular endothelial cells, it has been shown previously that G6PD influences eNOS activity by regulating substrate availability. Furthermore, gene transfer of G6PD to increase G6PD expression and activity in aortic endothelial cells resulted in enhanced NADPH levels, which in turn increased eNOS activity and NO generation measured as cGMP, nitrate, and nitrite levels (104).

**Hyperhomocysteinemia Polymorphisms.** Extensive epidemiological studies have indicated that elevated levels of homocysteine, a sulfur containing amino acid formed as an intermediary compound during methionine metabolism (8), are an independent risk factor for atherosclerosis and arterial thrombosis (8). The adverse effects of homocysteine are manifold but ultimately lead to endothelial dysfunction with associated platelet activation and thrombus formation (105). Although severe hyperhomocysteinemia can be caused by inborn errors of metabolism resulting - from cystathionine β-synthase deficiency. Elevation of homocysteine levels can be also due to homozygosity of a common 677C/T point mutation in the coding region of the methylenetetrahydrofolate reductase (*MTHFR*) gene. It may also be caused by nutritional deficiencies in the vitamin cofactors required for homocysteine metabolism (e.g. folate, vitamin B12, and vitamin B6). An inverse correlation between serum levels of these cofactors and homocysteine has been clearly documented (8, 106). Despite constituting the most common genetic cause of mild to moderate hyperhomocysteinemia, homozygosity for the MTHFR *677T* allele, which leads to the substitution of valine by alanine in a potential folate-binding site and thermolability of the enzyme, has not been clearly associated with atherothrombotic disease (107). Other studies have shown a 3-fold increase in risk of CAD is associated with the MTHFR *677TT* genotype; yet others found no association (108, 109). Brattstrom and colleagues pooled the results of 12 case-control studies and determined that individuals homozygous for the MTHFR *677T* allele generally had fasting homocysteine levels higher than in heterozygous or normal individuals. Nevertheless, 8 of these studies did not report an increased risk of cardiovascular disease associated with the homozygous MTHFR *677TT* genotype, and it was concluded that this polymorphism is a modest risk factor for arterial thrombosis. Similar results were obtained in a meta-analysis of 40 observational studies involving 11,000 CAD patients and 12,000 controls: the overall carriers were 677TT genotype. The heterogeneity of findings was interpreted as being secondary to the interaction of the MTHFR 677C/T polymorphism and folate status. Evidence from previous reports supports this hypothesis: Jacques and colleagues determined that among individuals homozygous for the MTHFR *677T* allele, homocysteine levels were only elevated when plasma folate concentrations were low (110). There was no significant difference in plasma homocysteine levels between *677TT* homozygotes who had normal folate concentrations and individuals with the *677CC* genotype (8, 110). Similarly, homozygotes with the lowest plasma folate concentrations had the highest homocysteine levels (111). Thus, folate levels can confound the interpretation of genotype association studies. Therefore folate supplementation might be a means of preventing atherothrombotic events in these patients. A second common variant in MTHFR 1298A/C is associated with decreased enzyme activity *in vitro* and *in vivo*, especially when occurring simultaneously with the 677 C/T polymorphism (112). Fasting homocysteine levels are significantly higher in individuals heterozygous for both substitutions compared with individuals who carry only the 677C/T variant (113). These polymorphisms are in linkage disequilibrium, rarely forming the MTHFR 677T/1298C haplotype, and their role in arterial thrombosis has not been studied (114).

**Polymorphisms in Fibrinogen.** Among the components of the coagulation system, elevated fibrinogen has been most consistently associated with arterialthrombotic disorders. Several mechanisms explain the association of increased fibrinogen with arterial thrombotic disease (115). In addition, high fibrinogen concentrations lead to the formation of a fibrin clot with thin and tightly packed fibers that has high thrombogenicity, possibly because the small pore size restricts access of fibrinolytic enzymes (116). Fibrinogen levels are strongly correlated with traditional vascular risk factors, including age, physical inactivity, hypertension, smoking, and features of the insulin resistance syndrome. Furthermore, fibrinogen is an acute-phase reactant, in part owing to its upregulation via activation of interleukin-6-responsive elements in the promoter of all 3 fibrinogen chains; the acute-phase response is strongly implicated in the development of arterial disease and may arise from e.g. viral infection, inflammatory stimuli, and smoking. Alternatively, elevated fibrinogen might reflect the inflammation associated with atherosclerosis rather than being a causal risk factor (117). Genetic factors are estimated to contribute to 50% of the total variability in fibrinogen levels. Several polymorphisms have been identified in the genes encoding the 3 pairs of fibrinogen polypeptide chains (α, β, and γ); however, because the synthesis of the β -chain is rate-limiting *in vitro*, most studies have focused on this gene. The main β-chain variants include the Arg448Lys, *Bcl*I, -148C/T, -455G/A (*Hae*III), and -854G/A polymorphisms. The promoter polymorphisms are in strong linkage disequilibrium with each other. The -455G/A and -854G/A substitutions are the most physiologically relevant, because the respective alleles have distinct nuclear protein-binding properties and, reporter gene studies in HepG2 cells showed an increased rate of basal transcription in the less common -*455A* and -*854A* alleles (118, 119). Of the β -chain polymorphisms, the -455G/A has been the most extensively studied clinically. The -*455AA* genotype is present in 10% to 20% of the population and is correlated with fibrinogen levels that are 10% higher than in individuals with the *GG* genotype. Nevertheless, the relation between the -455G/A variant and the risk of arterial thrombotic disease is controversial; only limited case-control studies have been performed (120, 121). In a pooled analysis of inherited hemostatic risk factors and the risk of acute MI, homozygosity for the fibrinogen -*455A* allele was significantly though only marginally associated with a decreased risk of MI (122). In addition, the -*455A* allele has been associated with the progression of atheroma (123). In a recent cohort of elderly stroke patients, the presence of the -*455A* allele was associated with a 2.5-fold increase in risk of multiple lacunar infarcts but not with large-artery strokes. It has been suggested that elevated fibrinogen levels might predispose to the development of thrombosis primarily in small arteries (124). The association of other β-chain polymorphisms and arterial thrombosis remains unclear. In addition to the β-chain polymorphisms, a variant in the α-chain codes for a Thr312Ala substitution within its carboxy-terminal end (125), a region important for factor XIII-dependent processes, including α/α-chain cross-linking. Clots generated *in vitro* in the presence of the Ala312 fibrinogen isoform have more extensive α-chain crosslinking and in consequence, thicker fibers (126). Although in 1 study the Ala312 variant had a gene dose-related influence on poststroke mortality rates in subjects with arterial fibrillation (127, 128). More recent studies indicate that this α-chain polymorphism has a more relevant role in the pathogenesis of venous thrombo embolism (129). Taken together, these results suggest that Ala312-induced changes in clot structure predispose to embolization in both the arterial and venous vascular systems.

It is concluded that genetic changes in dyslipidemia, paraxonase, glutathione peroxidase, nitric oxide synthase, tetrahydrofolate reductase, glucose-6-phosphate dehydrogenase, and glutathione S-transferase could play a significant role in the initiation and progression of CVD.
